# Supplementary material for: Development of ARCADIA: a tool for assessing the quality of peer-review reports in biomedical research
Source: BMJ Open. 2020 Jun 8;10(6):e035604. doi: 10.1136/bmjopen-2019-035604 (PMC7282387; doi:10.1136/bmjopen-2019-035604)
Supplement: Supplementary data [file bmjopen-2019-035604supp001.pdf]

Supplementary file 1. Survey questionnaires

1.1. Survey questionnaire for authors

Welcome to the survey!

Although the peer review process plays a key role in research dissemination, only limited research has been conducted so far in this field.

The objective of this survey is to investigate the perspectives of biomedical editors and authors towards the **quality of peer review reports**. We hope this work will help us to develop a new tool to assess the quality of a peer review report in biomedical research.

Knowing your expertise, we would be very grateful if you could answer a few questions and share your opinion. The survey will take approximately **10 minutes** to complete. Your participation in this study is completely voluntary. If you decide to participate, all your answers will be de-identified and stored in a secured repository at Universitat Politècnica de Catalunya, Barcelona-Tech (Spain). The de-identified data from this study will be shared on Zenodo repository. In case you opt out of sharing your data, you will still be able to participate in the study.

This survey has received ethics approval from the Research Ethics Committee of the Universitat Politècnica de Catalunya, Barcelona-Tech (Spain).

This study is part of the **Methods in Research on Research** (MiRoR) project, a joint doctoral training programme in the field of clinical research funded by Marie Skłodowska-Curie Action http://miror-ejd.eu/. The objective of MiRoR project is to train future generations of scientists in Research on Research, a new discipline aiming to promote research integrity increasing research value and reducing waste in health research.

This study is conducted by **Cecilia Superchi**, a PhD student at Universitat Politècnica de Catalunya, Barcelona-Tech and Université Paris Descartes, Sorbonne Paris Cité in collaboration with Prof. Darko Hren (University of Split), Prof. José Antonio Gonzalez (Universitat Politècnica de Catalunya) and Prof. Isabelle Boutron (Université Paris Descartes).

If you have any questions about this study or your rights as a participant, you may contact by email Cecilia Superchi, cecilia.superchi@upc.edu or Darko Hren, dhren@ffst.hr

Do you agree to take part in the study?

- ◯ Yes, I agree
- ◯ No, I do not agree

Do you agree to share your de-identified data?

- ◯ Yes, I agree
- ◯ No, I do not agree

MiRoR Methods in Research on Research

Definition of peer review report quality

The **quality of a peer review report** could be defined as "to what extent the peer review report helps editors to make a fair decision and authors to improve the quality of the submitted manuscript"

Do you agree with this definition?

- ◯ Yes
- ◯ No
- ◯ Partially

Please add your comments and ideas on how to improve the definition

MiRoR
Methods in Research on Research

Importance of the items to assess peer review report quality

The following items have been identified in a systematic review as possible quality components of a peer review report.

We are interested to know your opinion on the importance of these items, particularly whether the item should be included in a new tool assessing the quality of a peer review report.

Please rate the IMPORTANCE of each item in assessing the quality of a peer review report from 1 (not important) to 5 (very important).

We expect that for some items it will not be easy for you to make a clear decision about the importance of the item. In those cases we still invite you to offer your rating but you can elaborate on your decision. Furthermore we invite you to suggest potential improvements in wording of the items.

The reviewer comments on the **originality of the study**

Not important	Slightly important	Moderately important	Important	Very important
1	2	3	4	5
○	○	○	○	○

Please add any comments about your decision and/or wording of this item (not a mandatory field)

The reviewer comments on the **interpretation of the study results**

Not important	Slightly important	Moderately important	Important	Very important
1	2	3	4	5
○	○	○	○	○

Please add any comments about your decision and/or wording of this item (not a mandatory field)

The reviewer comments on the **general strengths and weaknesses of the study**

Not important	Slightly important	Moderately important	Important	Very important
1	2	3	4	5
○	○	○	○	○

Please add any comments about your decision and/or wording of this item (not a mandatory field)

The reviewer comments on the **strengths and weaknesses of study methods**

Not important	Slightly important	Moderately important	Important	Very important
1	2	3	4	5
○	○	○	○	○

Please add any comments about your decision and/or wording of this item (not a mandatory field)

The reviewer comments on the **appropriateness of the statistical methods**

Not important	Slightly important	Moderately important	Important	Very important
1	2	3	4	5
○	○	○	○	○

Please add any comments about your decision and/or wording of this item (not a mandatory field)

The reviewer comments on the **methodological quality (internal validity) of the study**

Not important	Slightly important	Moderately important	Important	Very important
1	2	3	4	5
○	○	○	○	○

Please add any comments about your decision and/or wording of this item (not a mandatory field)

The reviewer comments on the **applicability and external validity of the study results**

Not important	Slightly important	Moderately important	Important	Very important
1	2	3	4	5
○	○	○	○	○

Please add any comments about your decision and/or wording of this item (not a mandatory field)

The reviewer comments on the **presentation and organization of the manuscript**

Not important	Slightly important	Moderately important	Important	Very important
1	2	3	4	5
○	○	○	○	○

Please add any comments about your decision and/or wording of this item (not a mandatory field)

The reviewer comments on the **adherence of the manuscript to the reporting guidelines**

Not important	Slightly important	Moderately important	Important	Very important
1	2	3	4	5
○	○	○	○	○

Please add any comments about your decision and/or wording of this item (not a mandatory field)

The reviewer's comments are **structured and organized**

Not important	Slightly important	Moderately important	Important	Very important
1	2	3	4	5
○	○	○	○	○

Please add any comments about your decision and/or wording of this item (not a mandatory field)

The reviewer's comments are **clear and easy to read**

Not important	Slightly important	Moderately important	Important	Very important
1	2	3	4	5

Please add any comments about your decision and/or wording of this item (not a mandatory field)

The reviewer knows and understands correctly **the content of the manuscript**

Not important	Slightly important	Moderately important	Important	Very important
1	2	3	4	5

Please add any comments about your decision and/or wording of this item (not a mandatory field)

The reviewer's comments are **constructive**

Not important	Slightly important	Moderately important	Important	Very important
1	2	3	4	5

Please add any comments about your decision and/or wording of this item (not a mandatory field)

The reviewer's comments are **detailed and thorough**

Not important	Slightly important	Moderately important	Important	Very important
1	2	3	4	5

Please add any comments about your decision and/or wording of this item (not a mandatory field)

The reviewer uses a **courteous tone**

Not important	Slightly important	Moderately important	Important	Very important
1	2	3	4	5

Please add any comments about your decision and/or wording of this item (not a mandatory field)

The reviewer comments on the **relevance of the study**

Not important	Slightly important	Moderately important	Important	Very important
1	2	3	4	5
○	○	○	○	○

Please add any comments about your decision and/or wording of this item (not a mandatory field)

New items to assess peer review report quality

Are there any other items to assess the quality of a peer review report that you think should be included?

Please list them.

MiRoR Methods in Research on Research

Demographic characteristics

What is your gender?

- ◯ Woman
- ◯ Man
- ◯ Prefer not to answer
- ◯ Other (please specify)

What is your age?

What is the highest level of education obtained?

- ◯ Bachelor Degree
- ◯ Master Degree
- ◯ PhD
- ◯ MD or equivalent
- ◯ Prefer not to answer
- ◯ Other (please specify)

MiRoR Methods in Research on Research

Author´s characteristics

What is your job title at your institution?

- Researcher
- Assistant Professor
- Associate Professor
- Professor
- Other (please specify)

What type of institution are you affiliated at?

- Private University
- Public University
- Research Centre
- Other (please specify)

Where is the institution located?

- Europe
- North America
- South America
- Africa
- Asia
- Australia

MiRoR Methods in Research on Research

Author´s characteristics

How long have you been publishing scientific papers?

- <5 years
- 6-10 years
- 11-15 years
- 16-20 years
- >20 years

Do you also work as biomedical editor?

- Yes
- No

Are you involved in making decisions on the manuscripts received by your journal?

- Yes
- No

Study results and next step

Please check which of the following options you would be interested in

- I would be interested in receiving the results of the present study
- I would be interested in participating in the validation study of a new tool for assessing the quality of a peer-review report

Please write down your name and email address. Your data will be **EXCLUSIVELY** used for the option(s) which you have previously chosen.

Name

Email address

1.2. Survey questionnaire for biomedical editors

Welcome to the survey!

Although the peer review process plays a key role in research dissemination, only limited research has been conducted so far in this field.

The objective of this survey is to investigate the perspectives of biomedical editors and authors towards the **quality of peer review reports**. We hope this work will help us to develop a new tool to assess the quality of a peer review report in biomedical research.

Knowing your expertise, we would be very grateful if you could answer a few questions and share your opinion. The survey will take approximately **10 minutes** to complete. Your participation in this study is completely voluntary. If you decide to participate, all your answers will be de-identified and stored in a secured repository at Universitat Politècnica de Catalunya, Barcelona-Tech (Spain). The de-identified data from this study will be shared on Zenodo repository. In case you opt out of sharing your data, you will still be able to participate in the study.

This survey has received ethics approval from the Research Ethics Committee of the Universitat Politècnica de Catalunya, Barcelona-Tech (Spain).

This study is part of the **Methods in Research on Research** (MiRoR) project, a joint doctoral training programme in the field of clinical research funded by Marie Skłodowska-Curie Action http://miror-ejd.eu/. The objective of MiRoR project is to train future generations of scientists in Research on Research, a new discipline aiming to promote research integrity increasing research value and reducing waste in health research.

This study is conducted by **Cecilia Superchi**, a PhD student at Universitat Politècnica de Catalunya, Barcelona-Tech and Université Paris Descartes, Sorbonne Paris Cité in collaboration with Prof. Darko Hren (University of Split), Prof. José Antonio Gonzalez (Universitat Politècnica de Catalunya) and Prof. Isabelle Boutron (Université Paris Descartes).

If you have any questions about this study or your rights as a participant, you may contact by email Cecilia Superchi, cecilia.superchi@upc.edu or Darko Hren, dhren@ffst.hr

Aren't you a biomedical editor? Please take part in the survey for biomedical authors following this link: https://www.surveymonkey.com/r/REPORT_QUALITY_AUTHORS

Do you agree to take part in the study?

- ○ Yes, I agree
- ○ No, I do not agree

Do you agree to share your de-identified data?

- ○ Yes, I agree
- ○ No, I do not agree

Definition of peer review report quality

The **quality of a peer review report** could be defined as "to what extent the peer review report helps editors to make a fair decision and authors to improve the quality of the submitted manuscript"

Do you agree with this definition?

- Yes
- No
- Partially

Please add your comments and ideas on how to improve the definition

Importance of the items to assess peer review report quality

The following items have been identified in a systematic review as possible quality components of a peer review report.

We are interested to know your opinion on the importance of these items, particularly whether the item should be included in a new tool assessing the quality of a peer review report.

Please rate the IMPORTANCE of each item in assessing the quality of a peer review report from 1 (not important) to 5 (very important).

We expect that for some items it will not be easy for you to make a clear decision about the importance of the item. In those cases we still invite you to offer your rating but you can elaborate on your decision. Furthermore we invite you to suggest potential improvements in wording of the items.

The reviewer comments on the **originality of the study**

Not important	Slightly important	Moderately important	Important	Very important
1	2	3	4	5
○	○	○	○	○

Please add any comments about your decision and/or wording of this item (not a mandatory field)

The reviewer comments on the **interpretation of the study results**

Not important	Slightly important	Moderately important	Important	Very important
1	2	3	4	5
○	○	○	○	○

Please add any comments about your decision and/or wording of this item (not a mandatory field)

The reviewer comments on the **general strengths and weaknesses of the study**

Not important	Slightly important	Moderately important	Important	Very important
1	2	3	4	5
○	○	○	○	○

Please add any comments about your decision and/or wording of this item (not a mandatory field)

The reviewer comments on the **strengths and weaknesses of study methods**

Not important	Slightly important	Moderately important	Important	Very important
1	2	3	4	5
○	○	○	○	○

Please add any comments about your decision and/or wording of this item (not a mandatory field)

The reviewer comments on the **appropriateness of the statistical methods**

Not important	Slightly important	Moderately important	Important	Very important
1	2	3	4	5
○	○	○	○	○

Please add any comments about your decision and/or wording of this item (not a mandatory field)

The reviewer comments on the **methodological quality (internal validity) of the study**

Not important	Slightly important	Moderately important	Important	Very important
1	2	3	4	5

Please add any comments about your decision and/or wording of this item (not a mandatory field)

The reviewer comments on the **applicability and external validity of the study results**

Not important	Slightly important	Moderately important	Important	Very important
1	2	3	4	5

Please add any comments about your decision and/or wording of this item (not a mandatory field)

The reviewer comments on the **presentation and organization of the manuscript**

Not important	Slightly important	Moderately important	Important	Very important
1	2	3	4	5

Please add any comments about your decision and/or wording of this item (not a mandatory field)

The reviewer comments on the **adherence of the manuscript to the reporting guidelines**

Not important	Slightly important	Moderately important	Important	Very important
1	2	3	4	5

Please add any comments about your decision and/or wording of this item (not a mandatory field)

The reviewer's comments are **structured and organized**

Not important	Slightly important	Moderately important	Important	Very important
1	2	3	4	5

Please add any comments about your decision and/or wording of this item (not a mandatory field)

The reviewer's comments are **clear and easy to read**

Not important	Slightly important	Moderately important	Important	Very important
1	2	3	4	5

Please add any comments about your decision and/or wording of this item (not a mandatory field)

The reviewer knows and understands correctly **the content of the manuscript**

Not important	Slightly important	Moderately important	Important	Very important
1	2	3	4	5

Please add any comments about your decision and/or wording of this item (not a mandatory field)

The reviewer's comments are **constructive**

Not important	Slightly important	Moderately important	Important	Very important
1	2	3	4	5

Please add any comments about your decision and/or wording of this item (not a mandatory field)

The reviewer's comments are **detailed and thorough**

Not important	Slightly important	Moderately important	Important	Very important
1	2	3	4	5

Please add any comments about your decision and/or wording of this item (not a mandatory field)

The reviewer uses a **courteous tone**

Not important	Slightly important	Moderately important	Important	Very important
1	2	3	4	5

Please add any comments about your decision and/or wording of this item (not a mandatory field)

The reviewer comments on the **relevance of the study**

Not important	Slightly important	Moderately important	Important	Very important
1	2	3	4	5
○	○	○	○	○

Please add any comments about your decision and/or wording of this item (not a mandatory field)

MiRoR Methods in Research on Research

New items to assess peer review report quality

Are there any other items to assess the quality of a peer review report that you think should be included?

Please list them.

Methods in Research on Research

Demographic characteristics

What is your gender?

- ◯ Woman
- ◯ Man
- ◯ Prefer not to answer
- ◯ Other (please specify)

What is your age?

What is the highest level of education obtained?

- ◯ Bachelor Degree
- ◯ Master Degree
- ◯ PhD
- ◯ MD or equivalent
- ◯ Prefer not to answer
- ◯ Other (please specify)

MiRoR
Methods in Research on Research

Editor´s characteristics

What is your job title at your journal?

- Editor in chief
- Associate editor
- Academic editor
- Section editor
- Deputy editor
- Other (please specify)

Are you involved in making decisions on the manuscripts received by your journal?

- Yes
- No

At what type of journal do you currently working as editor?

- General Journal
- Specialty Journal

MiRoR
Methods in Research on Research

Editor´s characteristics

Where is the journal located?

- ◯ Europe
- ◯ North America
- ◯ South America
- ◯ Africa
- ◯ Asia
- ◯ Australia

How long have you been working as editor?

- ◯ <5 years
- ◯ 6-10 years
- ◯ 11-15 years
- ◯ 16-20 years
- ◯ >20 years

Does your work inside or outside the journal include authoring scientific papers?

- ◯ Yes
- ◯ No

MiRoR
Methods in Research on Research

Study results and next step

Please check which of the following options you would be interested in

- ◯ I would be interested in receiving the results of the present study
- ◯ I would be interested in participating in the validation study of a new tool for assessing the quality of a peer-review report

Please write down your name and email address. Your data will be **EXCLUSIVELY** used for the option(s) which you have previously chosen.

Name	
Email address	
