# Supplementary material for: Development of ARCADIA: a tool for assessing the quality of peer-review reports in biomedical research
Source: BMJ Open. 2020 Jun 8;10(6):e035604. doi: 10.1136/bmjopen-2019-035604 (PMC7282387; doi:10.1136/bmjopen-2019-035604)
Supplement: Supplementary data [file bmjopen-2019-035604supp002.pdf]

Supplementary file 2. Invitation email for corresponding authors and biomedical editors

From:
Cc:
To:
Subject: Academic Survey on Peer Review

Dear researcher,

As corresponding author of the article recently published in [CUSTOM 1], we would like to invite you to participate in an **academic survey**.

The objective of this survey is to investigate the perspectives of biomedical editors and authors on the **quality of peer-review reports**. We hope this work will help us to develop a new tool to assess the quality of a peer-review report in biomedical research.

The survey will take approximately **10 minutes to complete**. Participation in this study is completely **voluntary** and you may withdraw at any time.

This study is part of the **Methods in Research on Research** (MiRoR) project, a joint doctoral training programme in the field of clinical research funded by the European Union's Horizon 2020 Research and Innovation Programme under the Marie Sklodowska-Curie grant agreement No 676207 http://miror-ejd.eu/

We would be very grateful if you would take the time to complete our survey. **Your insights** as an author are **essential** to us.

If you have any questions, comments or queries please do not hesitate to contact us at cecilia.superchi@upc.edu or dhren@ffst.hr

We kindly thank you for your time, attention, and cooperation.

Sincerely,

**Cecilia Superchi**, PhD Student at Universitat Politècnica de Catalunya & Université Paris Descartes
**Darko Hren**, PhD, Prof. at University of Split
**José Antonio Gonzalez**, PhD, Prof. at Universitat Politècnica de Catalunya
**Isabelle Boutron**, MD, PhD, Prof. at Université Paris Descartes

From:
Cc:
To:
Subject: Academic Survey on Peer Review

Dear [Name] [Surname],

As [CUSTOM 1] at [CUSTOM 2], we would like to invite you to participate in an **academic survey on peer review.**

The objective of this survey is to investigate the perspectives of biomedical editors and authors on the **quality of peer-review reports**. We hope this work will help us to develop a new tool to assess the quality of a peer-review report in biomedical research.

The survey will take approximately **10 minutes to complete**. Participation in this study is completely **voluntary** and you may withdraw at any time.

You are also encouraged to **forward the link** of the survey to your colleagues who may be interested in participating in this study https://www.surveymonkey.com/r/REPORT_QUALITY_EDITORS

This study is part of the **Methods in Research on Research** (MiRoR) project, a joint doctoral training programme in the field of clinical research funded by the European Union's Horizon 2020 Research and Innovation Programme under the Marie Sklodowska-Curie grant agreement No 676207 http://miror-ejd.eu/

We would be very grateful if you would take the time to complete our survey. **Your insights** as a biomedical editor are **essential** to us.

If you have any questions, comments or queries, please do not hesitate to contact us at cecilia.superchi@upc.edu or dhren@ffst.hr

We thank you kindly for your time, attention, and cooperation.

Sincerely,

**Cecilia Superchi**, PhD Student at Universitat Politècnica de Catalunya & Université Paris Descartes
**Darko Hren**, PhD, Prof. at University of Split
**José Antonio Gonzalez**, PhD, Prof. at Universitat Politècnica de Catalunya
**Isabelle Boutron**, MD, PhD, Prof. at Université Paris Descartes
