# Supplementary material for: Development of ARCADIA: a tool for assessing the quality of peer-review reports in biomedical research
Source: BMJ Open. 2020 Jun 8;10(6):e035604. doi: 10.1136/bmjopen-2019-035604 (PMC7282387; doi:10.1136/bmjopen-2019-035604)
Supplement: Supplementary data [file bmjopen-2019-035604supp003.pdf]

Supplementary file 3. Top 30-biomedical journals with the highest impact factors

Full Journal Title**	IF
New England Journal Of Medicine*	79.3
Lancet*	53.3
JAMA-Journal Of The American Medical Association	47.7
BMJ-British Medical Journal*	23.3
JAMA Internal Medicine	20.0
Annals Of Internal Medicine	19.4
Nature Reviews Disease Primers	16.1
Journal Of Cachexia Sarcopenia And Muscle	12.5
Plos Medicine	11.7
Bmc Medicine*	9.1
Mayo Clinic Proceedings*	7.2
Cochrane Database Of Systematic Reviews	6.8
Journal Of Internal Medicine	6.8
Canadian Medical Association Journal*	6.2
Journal Of Clinical Medicine*	5.6
American Journal Of Medicine*	5.1
Translational Research*	4.9
Annals Of Family Medicine*	4.5
Medical Journal Of Australia*	4.2
American Journal Of Preventive Medicine*	4.1
Amyloid-Journal Of Protein Folding Disorders	4.0
Journal Of General Internal Medicine*	4.0
Deutsches Arzteblatt International	3.9
Palliative Medicine	3.8
Preventive Medicine*	3.5
British Medical Bulletin	3.4
European Journal Of Internal Medicine*	3.3
British Journal Of General Practice*	3.3
Journal Of Pain And Symptom Management*	3.2
Qjm-An International Journal Of Medicine	3.2

* Journal reporting the corresponding author in the PubMed abstract.
**Source: InCites Journal Citation Reports 2017 under the category “Medicine, general and internal”.
