# Supplementary material for: Development of ARCADIA: a tool for assessing the quality of peer-review reports in biomedical research
Source: BMJ Open. 2020 Jun 8;10(6):e035604. doi: 10.1136/bmjopen-2019-035604 (PMC7282387; doi:10.1136/bmjopen-2019-035604)
Supplement: Supplementary data [file bmjopen-2019-035604supp004.pdf]

Supplementary file 4. Complete participants characteristics

Characteristics	Editors<br>N=165
**Journal Role**	
Editor-in-Chief	50 (30.3%)
Associate Editor	63 (38.2%)
Academic Editor	7 (4.2%)
Section Editor	6 (3.6%)
Deputy Editor	12 (7.3%)
Other<br>(e.g. Statistical Editor, Patient Editor)	27 (16.4%)
**Involvement in making decisions on the manuscript**	
Yes	144 (87.3%)
No	21 (12.7%)
**Type of Journal**	
General Journal	39 (23.6%)
Specialty Journal	126 (76.4%)
**Authorship of scientific papers**	
Yes	141 (85.5%)
No	24 (14.5%)

Characteristics	Authors<br>N=224
**Occupation**	
Professor	63 (28.1%)
Associate Professor	31 (13.8%)
Assistant Professor	34 (15.2%)
Researcher	47 (21.0%)
Other<br>(e.g. Lecturer, Postdoc, PhD)	49 (21.9%)
**Type of Institution**	
Public University	134 (59.8%)
Private University	33 (14.7%)
Research Centre	17 (7.6%)
Other<br>(e.g. Hospital)	40 (17.9%)
**Employment as biomedical editor**	
Yes	63 (28.1%)
No	161 (71.9%)
**Involvement in making decisions on the manuscript**	
Yes	56 (88.9%)
No	7 (11.1%)
