# Supplementary material for: Development of ARCADIA: a tool for assessing the quality of peer-review reports in biomedical research
Source: BMJ Open. 2020 Jun 8;10(6):e035604. doi: 10.1136/bmjopen-2019-035604 (PMC7282387; doi:10.1136/bmjopen-2019-035604)
Supplement: Supplementary data [file bmjopen-2019-035604supp005.pdf]

Supplementary file 5. Codebooks

The first codebook is about the suggestions made by survey participants on how to improve the definition of peer review report quality we provided.

The second codebook is about the comments made by survey participants on importance and/or wording of each item (n=20).

The third codebook is about the identification of new items to assess peer review report quality by survey participants.

Codebook 1. Suggestions on how to improve the definition of peer review report quality

**Suggestions on how to improve the definition of peer review report quality (n= 87)**				
**Theme**	**Definition**	**Code**	**Sub-code**	**Example**
Assessment of different aspects of a study	Statements on the different aspects of a study that should be discussed in a peer review report	Accuracy of the study	NA	*Also helps ensure the accuracy of the content (at least in part)*
		Originality of the study	NA	*Should include recognition of what has already been done well where possible*
		Relevance of the study	NA	*Consider adding "the quality and value of the submitted manuscript." Or perhaps "quality and impact." The point is that some submitted work is high quality, but still not useful because it is just repeating prior work or answering an irrelevant question*
		Reproducibility of the study	NA	*The definition could broadly also take into account issues of reproducibility*
		Research integrity of the study	NA	*The definition should also include something about identifying plagiarism and conflict of interests*
		Robustness of the study	NA	*Not necessarily to improve the quality as that may be a secondary outcome. The peer review is to evaluate the scientific robustness of the research*

		Soundness of the study	NA	*Also - the extent to which the report critically assesses the soundness*
		Strengths and weaknesses of the study	NA	*I would include the extent to which the review accurately and clearly identifies weaknesses / limitations of the study ... but I don't disagree with your definition*
		Structure of the manuscript	NA	*A high quality peer review report may also identify the potential for a poorly structured paper to be revised into an impactful form*
		Study methodology and statistics of the study	NA	*Aspects such as rigorous statistical analysis and sampling/experimental design, degree of innovation, and the statement and testing of clear scientific hypothesis, should be addressed in a peer-review. Also, the technical issues and methodologies should be targeted during the peer-review process*
		Validity/trustworthiness of the study	NA	*What about the validity and trustworthiness of the findings?*
Consideration of journal´s policy	Statements on the consideration of the journal´s policy in writing a peer review report	NA	NA	*A good quality peer-review report takes into account journal policies and publication criteria while helping authors provide the best version of their work*
Irrelevant and ambiguous comments	Irrelevant and ambiguous comments for improving the provided definition of peer review report quality	NA	NA	*There is usually more than one report, so reportS*
Quality as a vague concept	Statements on the difficulty to define “quality”	Dependency on the type of journal and study	NA	*I would add at the end.... "based on a rubric specific to the type of article submitted"*
		Quality of research and quality of reporting	NA	*"Quality" is ambiguous. Relevant aspects of quality could include scientific validity (the extent to which the methods are adequate, the conclusions supported etc.) AND/OR reproducibility (the extent to which the study is described in sufficient detail that it could be reproduced). The former is a quality of the scientific study and the*

				latter is a quality of the text
Reviewer´s expertise	Statements on how the quality of a peer review report is related to the level of expertise of a reviewer	NA	NA	*The quality of the peer review also depends on how well the reviewer has understood the manuscript and the reviewer's level of expertise in the topic*
Reviewer’s comments characteristics	Statements on the different characteristics of a peer review report	Clarity of the comments	NA	*I think the definition should include whether the reviewers have expressed themselves clearly and unambiguously*
		Constructiveness of the comments	NA	*Perhaps this is implied in the proposed definition, but you could mention that a high-quality peer review includes constructive criticism -- that is, not just an identification of flaws but suggestions for remedies*
		Fairness and impartiality of the comments	NA	*Add: ‘is unbiased and competently-conducted’*
		Understanding correctly the content of the manuscript	NA	*The quality of the peer review also depends on how well the reviewer has understood the manuscript*
Role of external parties	Statements on the role of external parties in assessing the quality of a peer review report	NA	NA	*External parties should also play a role*
Scope of the peer review process	Statements on the scope of the peer review process	Different facets	NA	*Peer review has many facets*
		Ensuring accessibility to the readers	NA	*[..] and the accessibility to readers*
		Ensuring quality of science	NA	*I would like to insist on the role of peer-review to ensure the quality of the science presented in the manuscript*

		Evaluating rather than improving	NA	*The peer review is to evaluate the scientific robustness of the research*
		Independence	NA	*This definition does not capture the necessary independence of the peer review process*
		Related to decision making	NA	*This definition implies that editors' decisions can always override peer reviewers' appraisals. But an editor's appraisal of a paper should have equal weight to a peer reviewers appraisal*
		Transparency and critical appraisal	NA	*The focus of peer-review is transparency and critical appraisal. Peer review scope is broader than editorial decisions. Editorial decisions are a specific use case of peer review*
		Validation of the research	NA	*Peer review also helps to validate the research before publication, so the report also needs to do this to be effective*
		Variable process		*Peer review can be very variable; at its best it really improves the quality of papers. At its worst it is bullying and partial*
Timeliness of peer review process	Statements on the consideration of timeliness in defining the quality of a peer review report	NA	NA	*Timeliness should be included, less than 2 weeks is ideal*
Usefulness of the peer review report	Statements on the usefulness of a peer review report for authors and editors	Useful for authors	Improving manuscript quality	*It should be aimed at helping the authors improve the quality of their work.*
			More effective communication of research	*Peer review ideally contributes to effective communication through research publication, by exposing the author's work to the potential audience(s) for it and thus showing where readers stumble or identify limitations that need to be recognized. I think it would improve the definition if you could work in something about effective communication, which can be distinct from quality per se. More effective, clearer communication promotes more learning from the article and a stronger link to implementation. Also, quality should*

				probably be judged in terms of the purpose of the study; a delivery system study for example must provide more information on context in order to be useful than does a classical randomized trial such as of a pharmaceutical.
		Useful for editors	Filtering studies	*Also, 1) a check on poor research,*
			Decision to enhance the readership and citations	*helps editors to make a fair and informed decision that will enhance the readership and citations of the journal*
			Enabling fair decision	*I agree that they are primarily good to the extent that they help editors. "Fairness' is important, but enabling 'informed' and 'rigorous' decisions matter too.*
			Leading to incorrect decision	*Implies that the reviewer is making sensible suggestions, which may not be the case (and which an editor may not pick up on). E.g., a non-statistical reviewer commenting (incorrectly) on statistical methods and the editor is unaware if the comments are relevant/correct. The review could help the editor make a decision but it could be an incorrect decision.*
			Same weight for editors and reviewers appraisal	*This definition implies that editors' decisions can always override peer reviewers' appraisals. But an editor's appraisal of a paper should have equal weight to a peer reviewer's appraisal. This top down system can allow for bias.*
		Useful for both editors and authors	NA	*The point you suggest to help BOTH editor AND authors is a key element. When I ask for revision I provide new insights or suggestions to improve the quality and accuracy of a paper.*
Wording of the definition	Statements on how to improve the definition of the peer review report quality	Disagreement with the use of fair	NA	*This seems reasonable but I would leave out the word "fair" as I would assume that editors always aim to make fair decisions!*

Codebook 2. Comments on the importance and/or wording of each item

**Relevance (n = 56)**				
**Theme**	**Definition**	**Code**	**Sub-code**	**Example**
Author’s responsibility	Statements on the author’s responsibility to discuss the relevance of the study in the manuscript	NA	NA	*Relevance should be discussed by the authors and ultimately is decided by readers. I would expect reviewers to comment on the author's discussion on the relevance, and only exceptionally come with considerations of his own*
Contribution to the knowledge	Statements on the importance of the study as contribution to the scientific knowledge	NA	NA	*Relevance is important in the context of both the contribution to the knowledge base*
Dependency on the type of journal	Statements on how biomedical journals differently evaluate relevance of a study based on their own criteria	NA	NA	*Relevance also depends on the scope of the journal, and that is an editorial decision, opinion of the reviewer is not so important*
Editor’s responsibility	Statements on the editor’s responsibility to evaluate the relevance of the study	NA	NA	*This is largely an editorial decision*
Influencing editor’s decision	Statements on how the relevance of a study can influence an editorial decision	NA	NA	*This is very helpful for the Editor to make a decision on the manuscript*
Readers as final judges	Statements on how readers are the final judges of the relevance of a study	NA	NA	*Relevance should be discussed by the authors and ultimately is decided by readers*

Related to other items	Statements on the link of the item with other items	NA	NA	*I took this to be the same question as the earlier one on applicability*
Reviewer’s expertise	Statements on how the assessment of the relevance of a study depends on reviewer´s expertise	NA	NA	*Not important if you are a statistical reviewer, of a clinical article, without knowing the clinical area. BUT otherwise VERY IMPORTANT for clinical reviewers*
Subjective item	Statements on the subjective interpretation of the term “relevance”	External validity	NA	*Another aspect of relevance might relate to external validity or generalizability -- e.g., a lab study that does not have relevance to the real world*
		Future impact	NA	*Relevance may lie in the future, not in the present*
		Novelty	NA	*There are various aspects of "relevance" -- i.e., it might not be a significant contribution to the literature because the findings are not at all novel*
Wording of the item	Statements on how to improve the wording of the item	NA	NA	*Relevance to the broader field, or to general society?*

Originality (n= 56)				
**Theme**	**Definition**	**Code**	**Sub-code**	**Example**
Dependency on the type of journal	Statements on how biomedical journals differently evaluate the originality of a study based on their own criteria and policy	NA	NA	*Depends on the journal policy. More important when the reader is the client, less important when the author is the client*
Editor's responsibility	Statements on the editor's responsibility to evaluate the originality of the study	NA	NA	*Many journals mainly open which have different editorial policies now-a-days do not ask reviewers' to judge the originality. This is losing importance in open access era*
Importance of replication and confirmatory study	Statements on the importance of conducting replication and confirmatory studies	NA	NA	*Not always important to be original study as some are trying to duplicate findings from previous studies*
Importance of the item	Statements on the importance of the item in assessing the quality of peer review report	Slightly important item	NA	*This is only slightly important in that - once a study has been conducted*
		Important item	NA	*I find that important. The twentieth me too study is not relevant for the knowledge field*
Open access vs. subscription journal	Statements on how open access journals and subscription journals assess differently the originality of a study	NA	NA	*For our journal, this is very important, although I think for some others with a pay-for-publication model they place less importance on e.g. novelty*

Reviewer’s expertise	Statements on how the assessment of the item depends on reviewer´s expertise	Reviewer as not the best judge	NA	*Not all reviewers will necessarily be familiar with the literature in a particular area and may not be able to comment on originality of the study*
Wording of the item	Statements on how to improve the wording of the item and better define it	Impact rather than originality	NA	*Some less original studies can still be of significant value, so I prefer comments on impact to comments on originality*
		Originality as novelty	NA	*The Editor is usually aware of this, particularly Editors of journals with high impact factors, who are very keen to publish manuscripts reporting original/novel findings*
		Originality as what the study adds	NA	*I would prefer to think of this in terms of whether it really adds to our knowledge*

Interpretation of results<br>(n= 33)				
**Theme**	**Definition**	**Code**	**Sub-code**	**Example**
Avoiding exaggeration & misinterpretation and censoring divergent opinions	Statements on the importance of the item to avoid exaggeration and misinterpretation of study´s results	NA	NA	*This is an area where the reviewer may have a valuable role in tempering an author's enthusiasm, hubris or bias*
Conclusions supported by results, S&W and literature	Statements on the importance that study´s conclusions are supported by results, strengths and weaknesses and literature	NA	NA	*Interpretation of the findings should be judged by its coherence with findings and study limitations and strengths, and by its coherence with literature*
Contribution to the knowledge	Statements on the importance of the study as contribution to the scientific knowledge	NA	NA	*A judgement on the new contribution to knowledge*
Importance of the item	Statements on the importance of the item in assessing the quality of peer review report	Very important item	NA	*Interpretation of the results is crucial- it determines the message that is sent out. It is very important that reviewers pay attention to this interpretation*
Useful for readers	Statements on the uselessness of the item for the readers	NA	NA	*These are useless for the reader*
Liberty in the discussion section	Statements on the liberty of the authors to interpret study´s results in the discussion section	NA	NA	*As long as the results are crystal clear the authors can take some liberties in the discussion. As long as it is clear what is speculative*

Objective interpretation	Statements on the importance of the objectivity of the study´s interpretation	NA	NA	*As long as it is an objective interpretation without any confirmation bias*
Related to other items	Statements on the link of the item with other items	NA	NA	*See comments on methods above*
Related to study flaws	Statements on the importance of commenting on the item especially if there are major errors in the manuscript	NA		*This is important if there are any major flaws or if an alternative explanation for findings should be considered*
Related to study implications	Statements on the importance of commenting on the item for generating new hypothesis to test	NA	NA	*To me, this is the most important issue, for the point of papers is to generate new hypotheses to test. Unfortunately, in my field, editors often want to see the facts, but are wary about interpretations, probably about long-winded speculation in the past*
Reviewer’s expertise	Statements on how the assessment of the item depends on reviewer´s expertise	Statistics expertise	NA	*If the reviewer is experienced in statics can make a good interpretation of the results*
Rushed interpretation as common problem	Statements on the poor interpretation of the study´s results as common problem	NA	NA	*Discussion is the most important part of the manuscript. And sometimes it is a bit rushed by authors*
Scope of the PR process	Statements on the scope of the peer review process	Assisting editors to understand results	NA	*Editors aren't technical experts in every field. The PR process*

				is therefore important is assisting editors understanding the significance of results
Subjective item	Statements on the subjective interpretation of the term “interpretation of the results”	NA	NA	*All results are open to a variety of interpretations*
Wording of the item	Statements on how to improve the wording of the item and better define it	NA	NA	*The reviewer must comment on the discussion section, of which the interpretation is a part. But other elements (comparison with existing research etc.) is also important. I would replace 'interpretation' with 'discussion'*

Strengths and weaknesses (general)<br>(n= 21)				
**Theme**	**Definition**	**Code**	**Sub-code**	**Example**
Author’s responsibility	Statements on the author’s responsibility to discuss the strengths and weaknesses of the study in the manuscript	NA	NA	*The paper should, the reviewer only should if the paper is missing something important*
Important when manuscript is overly long	Statements on the importance of the item when the manuscript is overly long	NA	NA	*Important when a manuscript is overly long*
Specificity of the comments	Statements on the importance of the specificity of the comments	NA	NA	*Specificity is more important*
Related to other items	Statements on the link of the item with other items	NA	NA	*This is repeated above for methods. So these two components overlap. However, I mark this as important*
Related to the study	Statements on the importance of commenting on the strengths and weaknesses of the study´s aims and study´s flaws	Related to study aims	NA	*I think this needs to be specifically related back to clear study aims and objectives (perhaps this is a separate category? If not I think it should be). Even a beautiful study design with great validity and statistics is rubbish if it doesn't allow you to answer your research questions! I always look to see if the analyses and interpretation address the goals of the study*

		Related to study flaws	NA	*This is important where there are issues*
Reviewer’s expertise	Statements on how the assessment of the item depends on reviewer´s expertise	NA	NA	*Provided that the reviewer has the methodological skills to comment on methodological strengths and weaknesses.*
Taking into account reader’s perspective	Statements on the importance of taking into account the reader´s perspective by peer reviewers	NA	NA	*Peer reviewers should take the reader perspective and ensure the manuscript is well balanced on these*
Including S&W in the general comments	Statements on including strengths and weaknesses in the general comments	NA	NA	*These should be clearly identified in the general comments*
Useful for editors	Statements on the importance of the item for editors for making an editorial choice	To make a decision	NA	*Important for deciding to accept or reject a manuscript*

**Strengths and weaknesses (methods)<br>(n= 29)**				
**Theme**	**Definition**	**Code**	**Sub-code**	**Example**
Author's responsibility	Statements on author's responsibility to evaluate the item	NA	NA	*Authors should already be doing this*
Dependency on the methods quality	Statements on the importance of the item in relation to the quality of the methods of the study	NA	NA	*This really depends. If the methods are spurious, of course, this needs to be indicated*
Dependency on the type of journal and study	Statements on how the assessment of the item depends on the type of journal and study	NA	NA	*Methods are very important for our journal*
Focusing on the weaknesses	Statements on the importance of the item especially focusing on the weaknesses of the study	NA	NA	*Important, especially the weaknesses, where there is an obvious need*
Importance of the item	Statements on the importance of the item in assessing the quality of peer review report	Very important item	NA	*This is absolutely key to the interpretation of the study. Unfortunately most reviewers, in my field, do not fully understand current (and correct) methods*
Related to other items	Statements on the link of the item with other items	NA	NA	*Yes, but it is confusing to separate this from the general strength and weaknesses. The question should be if the reviewer thinks that the message can (potentially) answer the research question*

Reviewers' expertise	Statements on how the assessment of the item depends on reviewer´s expertise	NA	NA	*Providing the reviewer is suitably qualified to comment on the methods. In my experience, far too many reviewers comment on aspects of the Methods for which they are able to confidently comment on*
Subjective item	Statements on the subjective interpretation of the term "strengths and weakness (methods)"	To give indication on own stance	NA	*As long as the reviewer gives some indication of his/her own stance in determining strength and weakness*
Commenting on the transparency of methods used	Statements on the importance of commenting on the transparency of the methods used by the peer reviewers	NA	NA	*Authors should already be doing this. Is it necessary for reviewers to also state this, or is it redundant? Reviewers could comment on whether the authors have been transparent about the strengths and limitations*
Usefulness	Statements on the usefulness of the item for both editors and authors	Useful for authors	NA	*For the author if the strengths and weaknesses are not properly addressed in the paper*
		Useful for editors	NA	*This is important for the editor to make a decision*
Wording of the item	Statements on how to improve the wording of the item	NA	NA	*Appropriateness of methods (based on question) may be more important...unless there is a problem, then strengths and weaknesses becomes important*

Statistical methods (n=115)				
**Theme**	**Definition**	**Code**	**Sub-code**	**Example**
Dependency on the type of study	Statements on how the assessment of the item depends on the type of study	NA	NA	*For some articles (e.g. RCTs, meta-analyses, and observational studies) assessment of the statistical methods is important. For other types of articles (reviews, commentaries, editorials) this is of less importance*
Editor's responsibility	Statements on the editor´s responsibility to determine the necessity of a statistical review for a study	Employment of statistical assessors by journals	Statistics sub-editor	*Journal needs to engage a statistics sub-editor for that*
		No familiarity with some methods	NA	*Sometimes editor may not be as familiar with certain statistical methods which makes it more difficult to do this*
		Statistical support to the reviewers by journals	NA	*Journal editors should provide statistical support to reviewers*
		To determine the necessity of statistical review by journals	NA	*But I think that a lot (?) of reviewers are not sufficiently capable to do so. It might be more appropriate that the editor determines the necessity of statistical review and explicitly asks the reviewer if he/she is capable to do so*

No consensus on appropriateness of statistical methods	Statements on no consensus on the appropriateness of methods by peer reviewers	Variety of appropriate methods	NA	*Often there are multiple "appropriate" methods. It is important to use one of the appropriate methods*
Optional component of quality	Statements on the item as option component of the quality	NA	NA	*This should be an optional or "where relevant" component of the quality*
Related to other item	Statements on the link of the item with other items	NA	NA	*This belongs to assessing the methods. Should not be a separate item because there is qualitative research*
Reviewers' expertise	Statements on how the assessment of the item depends on reviewer´s expertise	At least one reviewer	NA	*At least one of the reviewers should have reasonable statistical knowledge*
		Content expertise	NA	*Many reviewers may be subject matter experts but not necessarily experts in statistics*
		General methods reviewer	NA	*This applies to the methods in general, whether or not they are statistical. I think at least one reviewer needs to comment on methods, but not every reviewer. Articles may not use statistics but an expert on the methods should review the article*
		Inappropriate advice	NA	*Non-statisticians should not be encouraged to comment on the statistical methods*

		Statistical reviewer	NA	*Statistical reviewer should do this*
		Commenting on own statistical expertise	NA	*In my view, the reviewer should be required to state whether or not she/he has the expertise to evaluate the statistical methods properly*
Commenting on the use of statistical methods	Statements on the importance to comment of the appropriate use of the statistical methods by authors	NA	NA	*And the use of stat. methods (some methods are used incorrectly by authors)*
Wording of the item	Statements on how to improve the wording of the item	NA	NA	*We get a lot of qualitative work so the key to this question is the appropriateness of the methods and then specifics based on type of methods*

**Methodological quality (n= 32)**				
**Theme**	**Definition**	**Code**	**Sub-code**	**Example**
Dependency on the type of study	Statements on how the assessment of the item depends on the type of study	NA	NA	*Where applicable it is important*
General comments	General statements	NA	NA	*But in any case, I think internal validity is very important -- if a study is claiming that there is a relation between two variables it should be on solid ground to do so*
Importance of the item	Statements on the importance of the item in assessing the quality of peer review report	Very important item	NA	*In my opinion this is the most important item*
Focusing more on methods than results	Statements on the importance of commenting on the methods	NA	NA	*In my opinion this is the most important item. I think a reviewer should primarily focus on methods rather than results*
Related to other items	Statements on the link of the item with other items	NA	NA	*I did not understand the difference between this question and the question "The reviewer comments on the strengths and weaknesses of study methods"*
Reporting of the study	Statements on the importance of good reporting for study reproducibility	NA	NA	*A description should be sufficient to repeat the study with a high likelihood to end up with the same results*

Reviewer’s expertise	Statements on how the assessment of the item depends on reviewer´s expertise	Acknowledgement lack expertise	NA	*Ideally yes. However good reviewers are also well aware of the limits of their own expertise. It is better for reviewers to acknowledge that they lack expertise in relation to a particular aspect of the methodology (this is OK - no one is expected to be an expert in everything) rather than attempt to critique something that is outside of their own scope of knowledge*
		At least one reviewer	NA	*At least one reviewer with expertise in methods should review the study*
		Technical vs. clinical expertise	NA	*When selecting reviewers one might choose some for technical expertise (methodology, statistics etc.) and others for clinical expertise/experience*
Making sure results are not biased	Statements on the importance of the study for making sure the study results are not biased	NA	NA	*Crucial to make sure the results are not biased*
Wording of the item	Statements on the wording of the item and how to improve it	Broad item	NA	*"Methodological quality" is a broad term that could apply to construct validity and statistical validity as well as internal validity*

**Applicability and external validity (n= 37)**				
**Theme**	**Definition**	**Code**	**Sub-code**	**Example**
Author´s responsibility	Statements on the responsibility of authors to comment on the applicability and external validity of the study providing sufficient information to the readers	NA	NA	*The paper should, not necessarily the reviewer*
Dependency on the practice of the reader	Statements on the difficulty to judge the importance of the item because it depends on the practice of the readers	NA	NA	*Difficult as would depend on the context of practice of the reader*
Dependency on the type of study	Statements on how the assessment of the item depends on the type of study	NA	NA	*It depends on the specific topic of the study*
Editor's responsibility	Statements on the editor's responsibility to evaluate the item	NA	NA	*This is more 'scope, which is for the editor to decide. But help from a reviewer is appreciated*
Helping the editor to understand reproducibility of the study	Statements on the importance of the item to decide if a study can be reproduced	NA	NA	*This is important when reporting novel findings as it helps the Editor to decide if the results can be reproduced by another group*
Importance of the item	Statements on the importance of the item in assessing the quality of peer review report	Less important item	NA	*This is of lesser importance. There should be sufficient information included for any*

				readers to come to this conclusion themselves
Related to other items	Statements on the link of the item with other items	NA	NA	*Similar to my answer about interpretation*
Related to the paper	Statements on the claims made in the paper by the authors and impact of the study	Future research	NA	*Applicability might lie in the future, not in the present*
		Impact	NA	*This is important only in relation to the claims made in the paper about the impact and implications of a study*
		Related to the claim & content of the paper	NA	*This depends on the claims made*
Reviewer's expertise	Statements on how the assessment of the item depends on reviewer´s expertise	Technical vs. clinical expertise	NA	*When selecting reviewers one might choose some for technical expertise (methodology, statistics etc.) and others for clinical expertise/experience*
Reviewers' comments characteristics	Statements on the different characteristics of a peer review report	Baring reviewers' opinion	NA	*This may be one area of the review where the reviewer can bring a personal opinion to bare. Does the reviewer think this is a useful paper?*
		Evidence based comments	NA	*Peer reviewers should provide citations (evidence) for their assessment. Simply saying that the results are not applicable to their practice is not enough*

		Tempering authors' enthusiasm	NA	*Similar to my answer about interpretation: this is an area where the reviewer may have a valuable role in tempering an author's enthusiasm, hubris or bias*
Subjective item	Statements on the subjective interpretation of the term "applicability and external validity"	NA	NA	*This can be very subjective and misleading*
Wording of the item	Statements on how to improve the wording of the item	NA	NA	*Applicability and external validity are two concepts, so this item is double-barrelled in not valid*

Presentation and organization<br>(n= 45)				
**Theme**	**Definition**	**Code**	**Sub-code**	**Example**
Dependency on the type of journal (and its policy)	Statements on how biomedical journals differently evaluate the item based on their own criteria	Presence of copy editors in the journal	NA	*Depends a bit on whether journals have good copy editors*
		Taking into account the average reader of the journal	NA	*The reviewer needs to take into account the "average reader" of the journal - will they understand the paper?*
General comments	General statements	NA	NA	*Peer review is not an editorial exercise, but clarity and reproducibility are part of good science*
Useful for editors	Statements on the usefulness of the item for editors	NA	NA	*Because the readability is important to those who've not seen it before. Especially helpful when a handling editor is new, I think.*
Importance of the item	Statements on the importance of the item in assessing the quality of peer review report	Less important item	NA	*This is less important, because as long as the content is there, a reader should be able to make use of the paper, even if it requires more effort. But if the presentation and organisation is really bad, then it needs to be addressed*
		Presentation more important than organization	NA	*Presentation is important organization is not*

Improving the manuscript	Statements on the importance of the item to improve the quality of the manuscript	Clear recommendations	NA	*Yes, but in a way that provides the authors with clear recommendations on how to make improvements. Design flaws cannot always be addressed after the study, but issues with presentation and organization of the manuscript can*
		Communication	NA	*Important when it will help improve the quality of the communication. Not necessary when it flows well*
		Readability	NA	*Important because this impacts readability*
Not going into irrelevant comments	Statements on the importance of not making useless comments	NA	NA	*Important when it will help improve the quality of the communication. Not necessary when it flows well.*
Formatting minutiae	Statements on peer reviewers focusing on minutiae	NA	NA	*Some reviewers focus on formatting minutiae*
Related to reporting guidelines	Statements on the link of the item with reporting guidelines	NA	NA	*I find reviewer comments on the presentation and organization of the manuscript moderately important if the manuscript follows a check list (e.g. STROBE) and/or standard formatting, and if is easy to understand and follow*

Responsibility	Statements on editor, author or reviewer´s responsibility to evaluate the item	Joint responsibility	NA	*I think this is the role of the editors as well as the reviewers.*
		Editor’s responsibility	NA	*Editors and editorial staff have a stronger role here.*
		Reviewer´s responsibility	NA	*I regularly make notes as to whether a section is better placed elsewhere in the document, and on sentence structure, and use and misuse of citations. I think this is an obligation that reviewers have to the author and the journal*
Subjective item	Statements on the subjective interpretation of the item	NA		*This is subjective and may vary between reviewers as log as general structure is preserved*
Wording of the item	Statements on how to improve the wording of the item	NA	NA	*The word "presentation" seems unclear. It could refer to the writing quality or to other factors*

Adherence to RG<br>(n= 73)				
**Theme**	**Definition**	**Code**	**Sub-code**	**Example**
Adherence to key points	Statements on the importance that a manuscript adherences on the key elements of a checklist	NA	NA	*I think whether a manuscript adheres to a specific item on a checklist is not that important. Adhering overall to the key elements needed to report is important*
Part of the PR process	Statements on the importance of checking the adherence of reporting guideline as part of the peer review process	NA	NA	*If it is widely accepted reporting guidelines like the Consort Guidelines I think that is an important part of peer review*
Author's responsibility	Statements on the author´s responsibility to follow reporting guidelines	Too demanding for authors	NA	*Some reviewers are too much strict on that*
Dependency on the type of journal	Statements on how the assessment of the item depends on the type of study	Consistent format	NA	*It would be great to have a consistent format and rubric to follow to increase comparability of manuscript and distress authors*
Dependency on the type of study	Statements on how the assessment of the item depends on the type of study	NA	NA	*Depends on type of study. For systematic reviews of course fundamental. For other studies this will be more and more important for easier comparisons between studies and for quality improvement. It makes our work easier if the*

				authors also compliance also improve
Editor’s responsibility	Statements on the editor’s responsibility to evaluate the item	Joint responsibility	NA	*The editor can also take care of this aspect*
		Pre-review	NA	*I believe this is the editor's job pre-review*
		Reformatting articles	NA	*We accept manuscripts that have been formatted for other journals for peer review. Of course we move towards acceptance they need to be reformatted*
General comments	General statements	NA	NA	*Universal reporting guidelines, like CONSORT, can be expected that all reviewers would know*
Importance of the item	Statements on the importance of the item in assessing the quality of peer review report	Important item		*Essential*
Lack of awareness	Statements on the lack of complete awareness about reporting guidelines from respondents	NA	NA	*In my experience, reviewers know little about the reporting guidelines of the journal for which they are reviewing. I think reviewers should always be sent details of the key guidelines. Otherwise they make criticisms or suggest changes which are incompatible with the guideline of the journal*

Providing reporting guidelines	Statements on providing reporting guidelines to reviewers	NA	NA	*The reviewer should receive the reporting guidelines applicable to the manuscript under review*
Reviewer’s responsibility	Statements on the reviewer´s responsibility to check if the manuscript adherences to reporting guideline	Less reviewer’s responsibility	NA	*I see that this is less the reviewer's responsibility to be honest*
		Making easier for reviewers	NA	*Important for improving standards in reporting, but this should be made as easy for the reviewer as possible, because otherwise it can be too arduous*
		Discussion of the study’s issues	NA	*Pointing out where the manuscript does not respect the guidelines is useful, but more important is discussing the issues themselves*
		Tedious for reviewers	NA	*When doing reviews, it is quite tedious to have to relate to difference reporting and formatting guidelines of particular journals*
Getting an accurate review	Statements on how reporting guidelines help delivering an accurate review	NA	NA	*That always irritating when authors do not follow the recommendations oto authors starting from pagination... which helps for delivering an accurate reviewing*

Unclear responsibility	Statements on the unclear responsibility of checking for the adherence of the manuscript to reporting guideline	NA	NA	*I am not sure whether this is the peer reviewers' or the editor's responsibility*
Utility of reporting guidelines	Statements on the uncertain utility of reporting guidelines	NA	NA	*I'm not convinced that reporting guidelines make that much difference, but they are certainly better than nothing*
Wording of the item	Statements on how to improve the wording of the item	Meaning of reporting guidelines	NA	*I simply do not know what this means. Which reporting guidelines?*

Structure of reviewer´s comments<br>(n= 33)				
**Theme**	**Definition**	**Code**	**Sub-code**	**Example**
Content and completeness are more important	Statements on the more importance of review´s content and completeness	NA	NA	*Completeness is more important than how structured*
Definition of structured and organized	Statements on how to define the item	NA	NA	*General comments (e.g. on style) followed by structured comments (line by line or section by section)*
Dependency on the structure of the manuscript	Statements on the importance of the item in relation to the structure of the manuscript	NA	NA	*Again - somewhat dependent on the structure of the manuscript that is being peer-reviewed*
General comments	General statements	NA	NA	*Peer review is not an editorial exercise, but clarity and reproducibility are part of good science.*
Useful for both authors and editors	Statements on the importance of the item in helping authors and editors	Making easier to answer	NA	*Makes it easier for the authors responding*
Importance of the item	Statements on the importance of the item in assessing the quality of peer review report	Not so important	NA	*It helps, but I'm not sure this is important enough to be assessed. Should covary strongly with other characteristics of the review*
Not related to meaningful content	Statements on the no relationship between a well-structured review and meaningful review's content	NA	NA	*Makes it easier to respond to but doesn't mean the review content is more or less meaningful*

Related to other item	Statements on the link of the item with other items	NA	NA	*Makes it easier to respond to comments if they are clear and easy to read*
Review reorganized by editors	Statements on the reorganization of a review by editors	NA	NA	*It is up to the editor to interpret the referee comments and make concrete recommendations or demands on the authors if needed.*
Reviewer as unpaid extra job	Statements on the voluntary job of reviewers	NA	NA	*Semantic point, be careful about asking too much from unpaid and unrewarded reviewers*
Standard structure of a review	Statements on the necessity to have a standard structure for reviews	Different perspective	NA	*Organised according to who's perspective...one person's structure is another's chaos?*
		More difficult for reviewers	NA	*But the more you set exacting standards for a review, the more difficult you make it for a reviewer. This is undoubtedly something to aim for, but reviewer time is an issue*
Time consuming to reorganize the review	Statements on the time lost in reorganizing the reviewer´s comments	NA	NA	*Otherwise time is lost in trying to reorganize and understand what the reviewer means*

Clarity (n= 26)				
**Theme**	**Definition**	**Code**	**Sub-code**	**Example**
Editors can make the comments clearer	Statements on editor´s task to edit the reviewer´s comments	NA	NA	*Helpful but not essential since the editor can help make sense of them for authors*
General comments	General statements	NA	NA	*Peer review is not an editorial exercise, but clarity and reproducibility are part of good science*
Useful for authors and editors	Statements on the usefulness of a clear peer review report for both authors and editors	NA	NA	*Otherwise neither the editor nor the authors can use the review appropriately*
Importance of the item	Statements on the importance of the item in assessing the quality of peer review report	Important item	NA	*Clarity is important*
		Less important	NA	*As long as the authors can understand the meaning, it is more important that the paper is clear*
Not a marker of quality	Statements on not considering clarity as marker of quality	NA	NA	*To me, although this is essential, it is more of an expectation of the review, rather than a marker of quality*
Reviewer as unpaid extra job	Statements on the voluntary job of reviewers	NA	NA	*But also conscious that we're all writing reviews late at night and so sometimes the ideal 'slips'*

To avoid repeated cycles of PR	Statements on the importance of the item to avoid repeated cycles of PR	NA	NA	*Yes - to avoid repeated cycles of peer review*
Useful for authors	Statements on the usefulness of the item for authors	Authors can ask for further clarity	NA	*It should be acceptable for authors to query reviewers' comments and ask for further clarity*
		Easy to respond	NA	*Makes it easier to respond to comments if they are clear and easy to read*
		Making sure the comments are intended	NA	*It is necessary to improve the chances that the comments are taken as intended*
Wording of the item	Statements on how to improve the wording of the item	Disagreement on the wording easy to read	NA	*I think easy to understand may be a better way to say this. I'm not sure easy to read is as applicable in the age of the computer*

**Constructiveness (n=46)**				
**Theme**	**Definition**	**Code**	**Sub-code**	**Example**
Clear guidance	Statements on the importance to give clear guidance on how to improve the manuscript	NA	NA	*Worth emphasising that they should, where appropriate, give clear guidance on how paper might be improved and not be derogatory*
Dependency on the paper quality	Statements on how the constructiveness of reviewer´s comments depends on paper quality	NA	NA	*I suppose there will be some submissions which are so poor, this will be difficult*
Extent of the comments	Statements on the consideration to what extent reviewer`s comments could be addressed	NA	NA	*I think that reviewers should also consider to what extent their comments can be addressed. For example, if it's a paper on a survey, it's not helpful for a reviewer to say that more people should be surveyed*
General comments	General statements	NA	NA	*The worst reviews are the ones where the reviewer just rambles on and does not provide something to respond to*
Importance of destructive comments	Statements on the importance of destructive comments	NA	NA	*Some bellicose reviews are pretty helpful*
Importance of the item	Statements on the importance of the item in assessing the quality of peer review report	NA	NA	*I would rank this as the most important*

Marker of quality	Statements on constructiveness as a marker of quality	NA	NA	*Constructiveness flags the reviewer's interest in improving the paper, so it is a marker of the likely value of their review*
Negativity of the comments	Statements on the total negativity of reviewer´s comments	NA	NA	*The reviewers' comments are important, however at times, without any reasons the comments are totally negative*
Not mandatory requirement	Statements on constructive comments as not a mandatory requirement	NA	NA	*They can be, but it's not mandatory. Some manuscripts shouldn't be published*
Not reviewers' responsibility	Statements on how reviewers should not rewrite the paper but be respectful	NA	NA	*It is not the reviewers' job to rewrite the paper or mentor the authors. However comments should always be respectful*
Related to author's experience	Statements on how constructive comments are related to the experience of authors	NA	NA	*It depends on the status of the author. A beginner in a field needs encouragement and support. An older expert who is talking rubbish deserves more direct language*
Related to recommendation	Statements on how constructive comments are also useful if the manuscript is rejected	NA	NA	*Important, even if the recommendation is to reject: the authors will probably submit elsewhere, the comments can be useful for them in order to improve the paper*
Related to the readership's interest	Statements on how constructive comments are related to the interest of readership	NA	NA	*Unless manuscript really not of interest to readership, then I would not expect a reviewer to*

				spend a lot of time essentially helping the authors
Related to other items	Statements on the link of the item with others	NA	NA	*Hands in hands with being courteous*
Sometimes difficult to be constructive	Statements on how comments are sometimes difficult to present in a constructive way	NA	NA	*I am not sure this reflects quality - valid concerns over methodology, results etc. are sometimes difficult to present in a constructive way. Clearly being constructive is preferable though*
Subjective term	Statements on the subjective interpretation of the term "constructiveness"	NA	NA	*I think this is a subjective term*
Usefulness for both authors and editors	Statements on how constructive comments are useful for both editors and authors	Useful for authors	NA	*Directly linked to helping the author improve the manuscript.*
		Useful for editors	NA	*In case the review aims to support the editor to offer a revision, constructiveness of the review is more relevant*
Wording of the item	Statements on how to improve the wording of the item	NA	NA	*I want respectful and helpful. Sometimes that is different than "constructive."*

Detail/Thoroughness (n= 62)				
**Theme**	**Definition**	**Code**	**Sub-code**	**Example**
Accommodating reviewer's comments	Statements on how authors accommodate reviewer´s comments	NA	NA	*On the other hand, authors often spend a lot of time with accommodating reviewer comments that were maybe not that relevant to start with. So there is a limit to how detailed and thorough is still helpful and the authors should have the right to reject some of the requests*
Dependency on the paper quality	Statements on how detailed comments depends on the quality of the paper	Detailed when paper is inadequate	NA	*Sometimes, where a paper is clearly inadequate producing a detailed report is necessary*
Detailed but not useful review	Statements on how detailed comments are not always useful	NA	NA	*They can be detailed but not useful--for example, when they concentrate on grammar and spelling*
For improving or rejecting the manuscript	Statements on the importance of detailed review to improve or reject a manuscript	NA	NA	*Sometimes the length of the comments is greater than the length of the manuscript. Peer reviewers should provide positive suggestions how the paper can be improved or rejected. The forma of the comments should be the same as the format of the responses, e.g. what I propose and why*

Importance of the item	Statements on the importance of the item in assessing the quality of peer review report	NA	NA	*Most important!*
Inconsistency in length	Statements on how peer review report are inconsistent in length	NA	NA	*The most disconcerting thing about reviews is inconsistency - when one is five pages long, the other five lines*
Not always necessary	Statements on how detailed comments are not always necessary	NA	NA	*Ideally but not always necessarily*
Providing a justification	Statements on the importance of providing a justification in the comments	NA	NA	*This is a pet peeve of mine. Some reviewers say things like "it has been demonstrated that this method of analysis is flawed" without providing a reference, for instance*
Related to other items	Statements on the link of the item with others	NA	NA	*I would have thought clarity was a more important criteria then being detailed but agree about thoroughness*
Reviewer as unpaid extra job	Statements on the voluntary job of reviewers	NA	NA	*Reviewers' time is valuable*
Straight to the critical points	Statements on the importance of succinct comments	Detecting fatal flaws	NA	*Focusing on one major flaw is more important than recitating all the typos*
		Excessive details	NA	*But they can be too detailed leading to a report that is too long overwhelming the author with too many requested revisions*

		Expectation from the authors	NA	*the most important is that the reviewer clearly indicate what he/she is expecting from the authors*
Wording of the item	Statements on how to improve the wording of the item	NA	NA	*"detailed, thorough and clear" (or unambiguous)*

Objectivity (n= 62)				
**Theme**	**Definition**	**Code**	**Sub-code**	**Example**
Citing own work	Statements on reviewers citing their own work in a peer review report	NA	NA	*Please can reviewers not cite their own work. This usually means they have approached the paper with bias*
Declaration of COI	Statements on the importance of reviewer’s conflict of interest declaration	More important than be objective	NA	*Declarations of competing interest and bias are more important than the claim to be objective*
Dependency on the study type	Statements on how objective comments are related to the type of study	Related to study’s quality	NA	*This is a fundamental principle, that the comments should be disinterested (i.e. not driven by the reviewer's self-interest) as this increases the chance of the comments relating to the paper's quality*
		Related to the novelty of the study	NA	*Moreover, reviewer's comments are strongly influenced by the reputation of the author and the novelty of the idea. The less known the author and the more novel the idea, the reviewer tends to be less objective*
Editor’s objectivity rather than reviewer’s objectivity	Statements on the importance of objectivity from editors rather than peer reviewers	NA	NA	*Editors are supposed to be objective, so reviewers can be opinionated if they wish*

Following a specific rubric	Statements on the importance to follow a specific rubric to guide comments by peer reviewers	NA	NA	*Reviewer should follow a specific rubric to guide comments and make revision manageable by author*
General comments	General statements	NA	NA	*This is one of the most critical elements of good peer review in my opinion but also one of the rarest things to find*
Importance of the item	Statements on the importance of the item in assessing the quality of peer review report	NA	NA	*This is a fundamental principle, that the comments should be disinterested (i.e. not driven by the reviewer's self-interest)*
Impossibility to be total objective	Statements on the impossibility for reviewers to be totally objective	Comments are subjective by definition	NA	*All reviews are subjective!*
		Desirable to express own opinion	Awareness of own experience	*A better expectation is that reviewers come to the role aware of their own backgrounds, culture, experiences, research and views on the topic will affect their assessment of the research*
		Reminding reviewers to be objective	NA	*As far as possible - reminding reviewers to be as objective as possible would be a good start*
		Subjective comments are helpful for both editors and authors	NA	*I think there are subjective comments that are still valuable to the authors and editors. For example, if the reviewer finds a section of the manuscript to be unclear, this is there subjective*

				opinion but can still help the authors re-assess and potentially improve that portion of the manuscript
Justification of the comments	Statements on the importance of substantiating the comments by peer reviewers	NA	NA	*It is important that the reviewer substantiates comments, and that the authors are able to respond in case of revising their manuscript, to the reviewer's comments*
Recruiting additional reviewers	Statements on recruitment of additional peer reviewers when the reviews are not objective	NA	NA	*When peer reviewers recommend citing own papers or clearly favour one treatment over the others, editors should recruit additional peer reviewers*
Related to authors' reputation	Statements on how peer reviewers are influenced by author's reputation	NA	NA	*Moreover, reviewer's comments are strongly influenced by the reputation of the author and the novelty of the idea. The less known the author and the more novel the idea, the reviewer tends to be less objective*
Related to other item	Statements on the link of the item with others	NA	NA	*Goes along with a courteous tone*
Reviewer's expertise	Statements on how the assessment of the item depends on reviewer´s expertise	NA	NA	*Every reviewer will always have their own perspective based on their expertise*
Wording of the item	Statements on how to improve the wording of the item	Unclear item	Difficult to define	*It would be very difficult to define this*

**Fairness (n= 55)**				
**Theme**	**Definition**	**Code**	**Sub-code**	**Example**
Biases are unavoidable	Statements on how reviewer's biases are unavoidable	NA	NA	*Reviewer biases are a reality, but one should (where possible) recognize them and phrase criticism in that light*
Editor's responsibility	Statements on the editor's responsibility to be fair	NA	NA	*Fairness is the editor's responsibility to judge*
Fair depends on author's characteristics	Statements on how the reviewer´s comments are influenced by author's characteristics	NA	NA	*It is well known that reviewers comments are not fair in terms of the location, ethnicity and gender of the authors*
Importance of the item	Statements on the importance of the item in assessing the quality of peer review report	NA	NA	*Fairness is extremely important*
Importance to back up opinions	Statements on the importance of backing up opinions by peer reviewers	NA	NA	*Crucial that a reviewer backs up their opinion with evidence from the paper/published literature*
Justification based on the paper quality	Statements on how justification of reviewers is based on the quality of the paper	NA	NA	*As per above, the reviewers comments must be justified based on the quality of the article rather than on their personal views*
Recognizing COI	Statements on recognition of reviewer´s conflict of interest	NA	NA	*More specifically, Editors should identify if the referee*

				has potential conflict of interest especially if he/she can have a conflict of interest working on the same field or topics. We all know such
Related to other items	Statements on the link of the item with others	NA	NA	*Objective, evidence-based, fair etc. are highly correlated*
Reviewers' perspectives	Statements on the importance of having peer reviewers with different perspective	NA	NA	*We often seek reviewers with different perspectives, so the entire editorial review team is constructed to be fair*
Subjective item	Statements on the subjective interpretation of the term "fairness"	NA	NA	*Fair, of course, is subjective*
Wording of the item	Statements on how to improve the wording of the item	Simplistic way to assess quality	NA	*Donald Trump sees the world in terms of fair or unfair. I think this is too simple a view*
		Unclear item	Difficult to define	*How do you define 'fair'?*
			Difficult to measure	*How would you measure "fairness" of a review?*

**Support by evidence (n= 69)**				
**Theme**	**Definition**	**Code**	**Sub-code**	**Example**
Context dependency	Statements on how the importance of item depends on the context	NA	NA	*Completely depends on the context; sometimes common sense can suffice but other times evidence-based critiques are necessary to show authors and editors why something needs changing*
Dependency on the type of study	Statements on how comments supports by evidence depends on the type of study	NA	NA	*Depends on the type of study*
Editor’s responsibility	Statements on the editor’s responsibility to determine if reviewer´s comments are relevant or not	NA	NA	*It is the editor's role to determine whether they are relevant or not*
Especially for supporting criticism	Statements on the importance of supporting criticism using evidence	NA	NA	*Comments, especially criticisms, should be supported by citations wherever possible; subjective criticism ("I prefer such and such a method ...") is not constructive*
Helpful when there are disagreements	Statements on the particular importance of the item especially when there are disagreements	NA	NA	*Particularly if authors objections are rooted in disagreements with their own opinions or collaborators opinions*
Importance of perceptions, opinions and experiences	Statements on the importance of perceptions, opinions and	Especially for too innovative manuscript	NA	*The exceptions are when the manuscript is too innovative or*

	experience of a reviewer in assessing a paper			*groundbreaking. In these case, knowledge and expertise to identify a possible major breakthrough is of utmost importance*
Importance of the item	Statements on the importance of the item in assessing the quality of peer review report	Important item	NA	*Very important. As an author and a frequent reviewer I have seen reviewer comments which are anything from completely wrong to simple statements of opinion without any evidence-base. But in my experience, what I would regard as quality reviews, up to date and accurate, are exceptionally rare*
		Not important item	NA	*Do you mean that they offer citations for their comments? If so, that's probably not important*
More information needed by authors	Statements on the necessity to get more information by authors	NA	NA	*Sometimes comments may be based on a hunch -- and more information from authors may be needed*
Unnecessary to provide evidence to each comments	Statements on how it is unnecessary to provide evidence for each comment	NA	NA	*I don't think reviewers need to cite something for every point that they make*
Related to other items	Statements on the link of the item with others	NA	NA	*If you mean, is it an objective review, then it is important*

Reviewer’s expertise	Statements on how the assessment of the item depends on reviewer´s expertise	NA	NA	*Based on the reviewer's knowledge and experience*
Selecting use of evidence	Statements on providing the use of no selective evidence	NA	NA	*Provided the use of evidence is not selective*
Difficulty of backing up all comments	Statements on the difficulty of backing up each comments	NA	NA	*Wild claims may need back up but it is unrealistic to expect reviewers to back up everything they say. A quicker system for raising and addressing queries would be a better response*
Wording of the item	Statements on how to improve the wording of the item	Difficult to measure	NA	*But I'm not sure how you judge this*
		Providing citations	NA	*Do you mean that they offer citations for their comments?*
		Unclear item	NA	*I don't really understand*

Knowledgeability<br>(n= 57)				
**Theme**	**Definition**	**Code**	**Sub-code**	**Example**
CoI between reviewers and authors	Statements on possible conflicts of interest between peer reviewers and authors	NA	NA	*Although this is very important it can create a conflict of interest as the authors and reviewers may be involved in the same field of research and this could result in a degree of bias for or against the research described in the manuscript*
General comments	General statements	NA	NA	*Reviewers should be able to commit time and effort to the process and be held accountable to the commitment.*
Importance of the item	Statements on the importance of the item in assessing the quality of peer review report	Important item	NA	*Obviously this is a key requirement*
Related to other items	Statements on the link of the item with others	NA	NA	*Peer reviewers should have understanding of research methodology as well*
Responsibility	Statements on editor or author´s responsibility to evaluate the item	Author's responsibility	NA	*Failures in this can be about whether the authors have communicated their work clearly*
		Editor's responsibility	NA	*They have to try to understand it - but sometimes they do not. This is where the editor must cast a critical eye to ensure the*

				reviewer has grasped the essence of the article.
Review as guide for editors	Statements on how a review is a guidance for editors		NA	*Extremely important. Nothing more annoying to an author than realising the reviewer has not fully read the paper. Also crucial if the review is to provide fair guidance for editors*
Reviewer as disadvantaged position	Statements on how the peer reviews is often in a disadvantaged position		NA	*The reviewer is often at a disadvantage as he/she is given limited information on which to make a decision on whether to accept or reject the offer to review*
Reviewer as unpaid extra job	Statements on the voluntary job of reviewers		NA	*Yes, but see issue above about late night reviewing*
Reviewers as readers proxy	Statements on how the reviewer acts as a proxy for the reader		NA	*The peer reviewer acts as a proxy for the reader, so a basic understanding of the manuscript's content is important*
Reviewers’ expertise	Statements on different reviewer´s expertise	Assessment reviewers’ expertise	NA	*This is very important. I've long thought that one of the review criteria should be self-ratings of the reviewer's expertise in the substantive and methodological aspects of the article*

		Declaration of competence by reviewers	NA	*Reviewers should declare their competence in the subject of the manuscript*
		More reviewers	NA	*Some reviewers know about methods and some about content. It would be ideal to always have both, but that is often not the case*
		Understanding also research methodology	NA	*Peer reviewers should have understanding of research methodology as well.*
Wording of the item	Statements on how to improve the wording of the item	Confusing item	NA	*I found this question confusing. Are you asking if the reviewer is competent to evaluate the content of the manuscript?*
		Difficult to assess	NA	*Not sure how you would know if the reviewer knows and understands correctly the content of the manuscript*
		Disagreement with the wording	NA	*"Knows" and "understands" are distinct concepts and should not be combined here*

Timeliness (n= 49)				
**Theme**	**Definition**	**Code**	**Sub-code**	**Example**
Better quality rather than on time	Statements on how a high quality review is more important than an on-time review	NA	NA	*But it is better to wait a while and have a high-quality review than to receive a quick, superficial and/or unfair review.*
Depends on the delay	Statements on how the importance of the item depends on the type of delay	NA	NA	*A few additional days of delay is not a major issue, while months of delay are*
Dependency on the type of journal	Statements on how biomedical journals differently evaluate the item	NA	NA	*Less important for pre-prints or F1000Research*
Difficult for editors	Statements on how long delay can cause difficulties to the editor	NA	NA	*Difficult for the editor if the delay is too long (or, worse, need to find another reviewer)*
Feasible and flexible deadlines	Statements on the importance to provide reasonable deadlines	Tendency to give short deadlines	NA	*Important, but there seems to be a trend among the editors to get reviews done in shorter amounts of time. Reminders are very helpful, but also some flexibility*
General comments	General statements	NA	NA	*The peer review process needs to be helpful for getting quality research into the public domain in a timely manner. It should not be a road block*
Golden rule	Statements on how to be on time is the golden rule	NA	NA	*It's the Golden Rule - it's just polite to be on time! Do as you would be done by etc.*

Importance of the item	Statements on the importance of the item in assessing the quality of peer review report	Important item	NA	*Very important for us as we try to provide a rapid response to the outcome of papers*
Journal´s reputation rather than good science	Statements on how the journal´s reputation is more important than good science	NA	NA	*Far too many editors now are asking for reviews to be complete in too little time. This is being done for the good of the journal's reputation, not for the good of science*
More time does not mean more quality	Statements on how giving more time does not means having more quality	NA	NA	*Increasing time for revision doesn't add anything to the quality*
Nor related to the quality of PR process	Statements on how the item is not related to the quality of the entire peer review process	NA	NA	*Again not sure this contributes directly to the quality of the peer review process but is important in terms of ensuring that publication timetables can be adhered to*
Orthogonal factors to review quality	Statements on orthogonal factors to review quality	NA	NA	*Availability, timeliness, reliability (reviewing when says will review) are orthogonal to review quality.*
Related to reviewer´s professionalism	Statements on how to be on time is related to reviewer´s professionalism	NA	NA	*This is more on the professionalism of the reviewer rather than the quality of the review*
Reviewer as extra unpaid job	Statements on the voluntary job of reviewers	Demanding work	NA	*There are heavy demands on our time. I find to carry out a quality review on a paper which needs*

				expert reviewing to achieve the two aims laid out initially, that it takes a minimum of half a day, and often a day to do a good job
		Difficult to find reviewers	NA	*We are all busy! biggest issue is finding someone to say yes in first place*
		Little delay	NA	*On time is pretty important but a little delay really is not a problem. I'm speaking as a journal editor... I always have plenty to do so a week delay is probably fine and will not cause authors too much pain*
		Reasonable time for the reviewer	NA	*But are the deadlines reasonable?*
Scope of the peer review	Statements on the scope of the peer review process	NA	NA	*Of course, with the caveat that peer review is voluntary and usually being fitted in around other work activities*
Time given by the journal	Statements on how journals give different deadlines	NA	NA	*Depends on how much time the journal gives. 2 weeks is not enough!*
Wording of the item	Statements on how to improve the wording of the item	Unclear item	NA	*Important to define what "on time" means. For example, is one day late a problem? Or a week late if the authors tell you?*

Tone (n= 40)				
**Theme**	**Definition**	**Code**	**Sub-code**	**Example**
Academia as though environment	Statements on the aggressiveness and competition in academia	NA	NA	*There is enough competition and aggressiveness in academia without having to receive reviews which are rude or condescending.*
Dependency on the author´s work	Statements on how the tone used by reviewers depends on the author´s work	NA	NA	*Depends on the author, and how much rubbish is being put forward*
Dependency on the paper quality	Statements on how the tone depends on paper quality	NA	NA	*And how much rubbish is being put forward*
Editor's responsibility	Statements on the editor´s responsibility to tone down the peer review reports	Removal comments by editors	NA	*Editor can tone down or edit out obnoxious comments, but it is better not to have to do this*
Golden rule	Statements on how to use a courteous tone is the golden rule	NA	NA	*Golden Rule again. Hiding rudeness behind anonymity is odious*
Hiding behind anonymity	Statements on how peer reviewers use anonymity to hide rudeness	NA	NA	*All too often, peer reviewers use the anonymity of the review process as an excuse to be rude and scathing in a way they would be unlikely adopt in person*
Impact of a rude review	Statements on how rude reviews can impact authors	NA	NA	*And if authors receive discourteous comments, this really does 'sour' the process*

				and put people off, especially new researchers
Importance of the item	Statements on the importance of the item in assessing the quality of peer review report	Important item	NA	*This is important. Especially to keep his/her nerves when looking at the first revised version and notice that the revision is not answering the queries and comments! Should also provide authors the keys to improve the paper and answer politely to referees...*
Not always a necessary requirement	Statements on how courteous tone is not always a necessary requirement	NA	NA	*This is nice, but not totally necessary*
Related to cultural differences	Statements on how courteous tone is culturally bound	NA	NA	*I think this is important, but courtesy is culturally bound*
Related to other items	Statements on the link of the item with others	NA	NA	*This relates to constructiveness, above*
Requirement	Statements on the requirement to use a courteous tone in a peer review report	NA	NA	*But it's an ethical requirement, and helps improve everyone's experience*
Review quality is important than courteous tone	Statements on how an higher quality review is more important than a courteous review	NA	NA	*Would you rather be treated by skilled, but rude, surgeon, or by a courteous flop?*
Useful for authors	Statements on the usefulness of the item for the authors	NA	NA	*The reviewer's aim should be to give comments that make the next version of the*

				manuscript better (whether or not it is accepted for that specific journal)
Wording of the item	Statements on how to improve the wording of the item	Difficult to define	NA	*It is impossible to define 'courteous' so I doubt that this is operationalisable*

Codebook 3. Identification of new items to assess peer review report quality

**Identification of new items to assess peer review report quality (n=152)**				
**Theme**	**Definition**	**Code**	**Sub-code**	**Example**
**Characteristics of reviewer's comments**	Statements on the characteristics of the comments made by a peer reviewer	Clarity	Clarity of the expected changes	*Being clear about the changes they want to see (vs. vague comments about weaknesses - what would most strengthen)*
			Clarity of the language	*The peer review report should be comprehensive and written clearly. It should not be ambiguous.*
		Constructiveness	NA	*Constructive attitude should include orientation and suggestion to authors to improve the manuscript.*
		Detailed	NA	*Specific details for concerns and suggestions for improvement are the keys for me. I need substantive concerns.*
		Evidence-based	NA	*A good peer-review report also includes references*
		Fairness/Unbiased	NA	*The comments should not only be fair, but also unbiased.*
		Specificity	NA	*I think that the challenge is that the comments are context*

				specific. Reviews are helpful when they identify something that is a challenge or something that can be done better. These rely upon the context.
		Structure of the peer review report	Additional comment to the editor	*Provides additional comments to the editor that provides context to the reviewer's assessment.*
			Explicit recommendation	*The reviewer makes an explicit recommendation about what decision to make – i.e., "reject", "revise and re-review", "accept", etc.*
			Initial summary	*The reviewer should begin her report with a short synthesis of the study (goals and main results)*
			Length of comments	*Length, very short peer review reports make me suspicious that they have even read the paper in enough detail. E.g. I once received a peer review "report" that had a single sentence along the lines of: "Good methods and results".*
		Tone	NA	*Candid*
**Related to peer review process**	General statements on the peer review process	Anonymity _PR process type	NA	*Reviewers and authors should be anonymous on both sides*

		Dependency on the type of journal	NA	*Reviewers should understand the nature of the journal that the manuscript had been submitted to - and should be prepared to state if he / she thinks the paper is not appropriate or relevant to that journal's readership.*
		Disclosure of reviewer's COI	Editor's task	*Conflict of interest. We often can tell that the journal has sent our paper to a reviewer who will not be objective in their review, and sometimes even when we've asked the editor to not use a particular reviewer. Editors have an obligation to insure a fair review, and often they do not. In these instances, the outcome is a foregone conclusion*
			Requests motivated by reviewer's COI	*The reviewer does not make requests that seem to be motivated by a competitive attitude or a conflict of interest.*
			Reviewer's publication record	*Publication record of the reviewer*
		Editor's responsibility	Balanced and fair decision	*Editor' decisions should also be balanced and fair, especially when reviewing are*

				discordant... Editors should also read papers... .
			Filtering reviews	*Editors need to protect authors from poor reviewers.*
		High profit of scientific publishing industry	NA	*The scientific publishing industry makes very high profits, in fact it is the most profitable "legal business model" among all economic activities.*
		Peer reviewers’ training	NA	*A good reviewer needs to be trained: should be important to organize courses*
		Poor quality of the second review	NA	*Completion of a second review after the first draft-this is often poorly done*
		Quality scale	NA	*It might be helpful to consider one of the research quality ratings scales that are used in quantitative reviews.*
		Review quality as usefulness to make an editor’s decision	NA	*The quality of a peer-review report that an author receives is partially determined by what the editors contribute to it before sending it to the author and how quickly they use it to make a decision. Too many don't send enough feedback, especially when two or more reviewers disagree.*

				This leads to three, four, or more back-and-forth "reviews" where reviewers are trapped in a cycle of disagreement and the editors won't make any significant contributions or a decision to resolve the disagreement.
		Reviewer as unpaid extra job	NA	*Reviewers are scientists that perform a professional service for the scientific publishing industry that in the vast majority of the cases is not paid.*
		Reviewer's final choice	Difference between major and minor revisions	*Always a grey area between Major revisions and Minor revisions that foxes a reviewer*
			Explanation choice	*If the reviewer makes a recommendation, e.g. accept or reject, they must provide a reason why. A review that just says "accept", "good work", is not a valid peer review.*
		Reviewer's recognition	Professional evaluators by publishers	*Alternatively, publishers may turn to "professional evaluators", who they may find in consultancy firms (KPMG, McKinsey, etc.) and pay their fees...........*
			Rewards for reviewers	*Payment for the reviewers should be considered, and this*

				would also increase the quality of the evaluations
		Saturation of the system	Professional evaluators by publishers	*So far reviewers are working "pro bono", and with the tremendous growth in the number of journals and the overall increase of the scientific activity worldwide (think just of the soaring number of papers coming from China in recent years) the system is becoming saturated, and reviewers becoming fed up*
		Scope of review	NA	*The number of items listed is a factor. If too many and in too minute detail, the article could be suppressed by the sheer workload of trying to address the comments*
		Weighting reviewer's comments	NA	*It is important for the ae to weight reviewers comments - some are rubbish and can be disregarded*
**Related to the study**	Statements on different aspects of a study that should be commented in a peer review report	About references	Suggesting relevant references	*Including references not known to the author*
		Addressing study's aims	NA	*I think the 'does this study address its stated aims' issue that I raised in my earlier responses is very important*

		Adherence to ethical guidelines	NA	*Comment on the study's adherence to ethical guidelines*
		Appendices	NA	*Use of supplemental material/appendices when appropriate (e.g., sensitivity analyses)*
		Applicability of the study	NA	*And general applicability*
		Authors' contribution and acknowledgments	NA	*Clearly articulate the role of every team member, and their contribution to the study. For evidence syntheses, require librarian involvement and give them authorship, the same with statisticians. Everyone in the team, without whose knowledge the study would not be possible, sound, or complete, should be acknowledged.*
		Context of the study	NA	*Puts the study in appropriate context*
		Data availability and software	NA	*Referees check the data availability and if new software actually works*
		Data quality	NA	*Quality of the data is most important*
		Ensuring disclosure of COI	NA	*Conflict of interests could be included*

		Ensuring inclusion of data sharing statements	NA	*Reviewers should ensure data sharing statements are included*
		Ensuring language quality	NA	*Comment on readability*
		Ethics	NA	*Ethical considerations of research*
		Importance of methods	NA	*Perhaps reviewing upto methods and evaluating the study that way is worth more consideration.*
		Literature is adequately reviewed	Most recent research	*Reviewer rating of whether The authors discuss the most recent relevant research on the topic*
		Originality	NA	*The added value of the study to what is already known.*
		Potential impact	NA	*Potential impact of study*
		Presentation (tables and figures)	NA	*And appropriateness of accompanying visual aids (graphs, tables e.t.c.).*
		Publication study’s protocol and deviation from it	NA	*Whether a protocol was lodged in publication or on an independent site e.g. OSF and whether it matches the paper and if not, if reporting of deviations is transparent.*

		Relevance	NA	*relevance*
		Replicability/Reproducibility	NA	*Whether the study can be replicated on current methods whether limitations are acknowledged (this was covered actually I think)*
		Study conclusions	NA	*And finally if the conclusion answers the research question.*
		Study introduction	NA	*If the in introduction leads to the research question*
		Study limitations	NA	*Whether limitations are acknowledged*
		Study theoretical framework	NA	*Logic of the theoretical framework*
		Study weaknesses	NA	*Reviewer comments on the limitations of the study*
		Suggestions for future studies	NA	*Suggestions for future studies*
**Reviewer’s expertise**		Considering reviewer’s expertise	NA	*I have experienced vastly different qualities of reviews, so I think that the reviewer's expertise in the area of the article needs to be considered.*
		Focus on the points suggested by editor	NA	*Suggestions from editor re: which points/themes to focus on*

		Knowing dimensions not assessed by reviewers	NA	*What the reviewer feels they cannot comment on (e.g. is outside their expertise)*
		Rating or commenting on own level of expertise	NA	*The reviewer should state those aspects of the study for which they have limited knowledge*
		Reviewer's type	NA	*Items need to be tailored for whether the reviewer is a stats (methodology) reviewer or clinical expert*

NA= not available
