# Supplementary material for: Development of ARCADIA: a tool for assessing the quality of peer-review reports in biomedical research
Source: BMJ Open. 2020 Jun 8;10(6):e035604. doi: 10.1136/bmjopen-2019-035604 (PMC7282387; doi:10.1136/bmjopen-2019-035604)
Supplement: Supplementary data [file bmjopen-2019-035604supp006.pdf]

Supplementary file 6. New items suggested by survey participants

New items	Example
1. Adherence to ethical guidelines	*"Comment on the study's adherence to ethical guidelines"*
2. Author's contribution and acknowledgements	*"Clearly articulate the role of every team member, and their contribution to the study. For evidence syntheses, require librarian involvement and give them authorship, the same with statisticians. Everyone in the team, without whose knowledge the study would not be possible, sound, or complete, should be acknowledged."*
3. Data availability	*"Referees check the data availability and if new software actually works"*
4. Disclosure of COI	*"Conflict of interests could be included"*
5. Data sharing statements	*"Reviewers should ensure data sharing statements are included"*
6. Study protocol	*"Whether a protocol was lodged in publication or on an independent site e.g., OSF and whether it matches the paper and if not, if reporting of deviations is transparent."*
7. Addressing study aims	*"I think the 'does this study address its stated aims' issue that I raised in my earlier responses is very important"*
8. Study introduction	*"If the in introduction leads to the research question"*
9. Study limitations	*"Whether limitations are acknowledged"*
10. Study conclusion	*"And finally if the conclusion answers the research question."*
11. Theoretical framework	*"Logic of the theoretical framework"*
12. Relevant literature	*"Reviewer rating of whether The authors discuss the most recent relevant research on the topic"*
13. Reproducibility	*"Whether the study can be replicated on current methods"*
