# Supplementary material for: Development of ARCADIA: a tool for assessing the quality of peer-review reports in biomedical research
Source: BMJ Open. 2020 Jun 8;10(6):e035604. doi: 10.1136/bmjopen-2019-035604 (PMC7282387; doi:10.1136/bmjopen-2019-035604)
Supplement: Supplementary data [file bmjopen-2019-035604supp007.pdf]

Supplementary file 7. Explanations of the items included in the ARCADIA tool

Domain 1: Importance of the study

Item 1.a Contribution

A study can contribute to scientific knowledge in many ways: it can be a novel or confirmatory study with little or great impact on society and/or the research community. The contribution of a study is therefore not only associated to its novelty. Studies also need to be replicated in order to verify the validity of their results. The peer reviewer should discuss the importance of the study's research question.

Item 1.b Relevant literature

The peer reviewer should check if the authors reviewed the relevant research related to the study's topic in order to situate the study within the context of the existing literature.

Domain 2: Robustness of the study methods

Item 2.a Study methods

The peer reviewer should evaluate the soundness of the study methods, such as the selection of the study design, assessment of the risk of bias, etc., to understand whether the methods were appropriate to the study's aims, as well as if they were properly used and reported.

Item 2.b Statistical methods

Data can be analysed in many ways, but the only appropriate statistical models are those that fit well with the study design and the characteristics of the variables. The peer reviewer with expertise in statistics should assess whether or not the study followed a suitable statistical procedure, as well as if they were correctly conducted and reported.

Domain 3: Interpretation and discussion of the study results

Item 3.a Study conclusions

The reviewer should verify if the study conclusions answer the research question(s) and correctly summarize the study results.

Item 3.b Study limitations

The reviewer should check if the weaknesses of the study are correctly identified and discussed in order to interpret the validity of the research.

Item 3.c Applicability and generalizability

The reviewer should comment on the applicability and generalizability of the study results. Applicability and generalizability are two underlying concepts of external validity [1]. The first concerns how "the results from a sample can be extended to the population from which the sample was drawn", while the second how "the inferences drawn from study participants can be used in the care of patients drawn from any populations" [1].

Domain 4: Reporting and transparency of the manuscript

Item 4.a Study protocol

Public access to study protocols is important to increase transparency and reduce waste of biomedical research. In the case of previous publication and/or inclusion as an additional file of a study protocol, the reviewer should verify that the major deviations from it are reported in the manuscript.

Item 4.b Reporting

The reviewer should comment if the reporting of the study is clear, complete and transparent enough for facilitating its reproducibility by verifying the adherence of the manuscript to the corresponding reporting guideline. The Enhancing the Quality and Transparency of Health Research (EQUATOR) Network provides a toolkit to be used during the peer review process for selecting the appropriate reporting guideline [2].

Item 4.c Presentation and organization

The reviewer should discuss the quality of the written language used in the manuscript, as well as of how the study results are presented (tables, figures, etc.).

Item 4.d Data availability

When applicable, the reviewer should ensure that the data and materials (e.g., dataset, software codes), supported the results reported in the manuscript, are available.

Domain 5: Characteristics of the reviewer's comments

Item 5.a Clarity

A peer review report should be clear (meaning that readers can easily understand its content), succinct and well organized (following the manuscript sections and, when it is necessary, providing line and page numbers) in order to be understood correctly by editors and authors.

Item 5.b Constructiveness

A peer review report should contain constructive and polite comments that allow the authors to improve the quality of their work and editors to take a decision.

Item 5.c Objectivity

Comments provided in a peer review report should be as objective as possible and, if considered appropriate, include references to support the reviewer's statements.

References

1. Murad MH, Katabi A, Benkhadra R, Montori VM. External validity, generalisability, applicability and directness: a brief primer. BMJ Evid-Based Med. 2018;23(1):17–9.
2. EQUATOR Network. Welcome to our toolkit for peer reviewing health research! [Internet]. Available from: https://www.equator-network.org/toolkits/peer-reviewing-research/
